# Impact of Delivery Mode on Neonatal Outcomes in Extremely Preterm Infants Born at 22 + 0 to 25 + 6 Weeks of Gestation

**DOI:** 10.3390/medicina61101880

**Published:** 2025-10-20

**Authors:** Filiz Markfeld-Erol, Martin Kuntz, Valeria Laufs, Susanne Tippmann, Ingolf Juhasz-Böss, Annette Hasenburg, Joscha Steetskamp

**Affiliations:** 1 Department of Obstetrics and Gynecology, Medical Center University of Freiburg, 79106 Freiburg, Germany; ingolf.juhasz-boess@uniklinik-freiburg.de; 2 Department of General Pediatrics, Adolescent Medicine and Neonatology, Medical Center University of Freiburg, 79106 Freiburg, Germany; martin.kuntz@uniklinik-freiburg.de; 3 Department of Obstetrics and Gynecology, University Medical Center, Johannes Gutenberg University Mainz, 55131 Mainz, Germany; valeria.laufs@unimedizin-mainz.de (V.L.); annette.hasenburg@unimedizin-mainz.de (A.H.); joscha@steetskamp.de (J.S.); 4 Department of Neonatology, Children’s Hospital, University Medical Center, Johannes Gutenberg University Mainz, 55131 Mainz, Germany; susanne.tippmann@unimedizin-mainz.de

**Keywords:** mode of delivery, preterm birth, cesarean section, vaginal delivery, intraventricular hemorrhage, necrotizing enterocolitis

## Abstract

*Background and Objectives*: Extremely preterm infants (<28 weeks’ gestation) face high risks of morbidity and mortality, and the optimal mode of delivery for this population is debated. This retrospective study evaluated the impact of delivery mode (vaginal vs. cesarean section) on neonatal outcomes in extremely preterm infants. *Materials and Methods*: Ninety-two singleton births at 22 + 0 to 25 + 6 weeks of gestation were analyzed. Primary endpoints were survival to discharge; intraventricular hemorrhage (IVH); necrotizing enterocolitis (NEC); and arterial umbilical cord pH. Secondary endpoints included APGAR scores; preterm premature rupture of membranes (PPROMs); and pathological vaginal microbial colonization. *Results*: Survival to discharge did not differ significantly between vaginal delivery (84.8%) and cesarean section (93.5%) (*p* = 0.140). No significant differences were observed for NEC, APGAR scores, or umbilical arterial cord pH. IVH occurred more often after vaginal birth (47.8% vs. 30.4%, *p* = 0.080). In multivariable analysis, male sex was significantly associated with adverse outcome (*p* = 0.041); while PPROM showed a borderline association (*p* = 0.079). Complete antenatal corticosteroid administration was more frequent in the cesarean group (*p* = 0.021) and represented a relevant confounder. *Conclusions*: Delivery mode had no significant effect on survival in this cohort, though IVH tended to occur more frequently after vaginal birth. Male sex and complete antenatal corticosteroid exposure were key determinants of neonatal outcome. Prospective studies are needed to establish evidence-based recommendations.

## 1. Introduction

Extremely preterm infants, defined as those born before 28 + 0 weeks of gestation, account for approximately 5% of all preterm births [1]. They face the highest risk for severe short- and long-term complications, including intraventricular hemorrhage (IVH), necrotizing enterocolitis (NEC), bronchopulmonary dysplasia (BPD), and respiratory distress syndrome (RDS) [2]. Gestational ages between 22 + 0 and 25 + 6 weeks represent the threshold of viability and pose particular challenges for perinatal care [3]. Within this critical window, both obstetric and neonatal management strongly influence outcomes.

The choice of delivery mode depends on multiple maternal and fetal factors, such as fetal presentation, estimated weight, maternal comorbidities, and anticipated neonatal prognosis [4,5]. However, the optimal mode of delivery for extremely preterm infants remains uncertain. Cesarean delivery has been associated with reduced mechanical stress on the immature brain and a potential decrease in the incidence of severe intraventricular hemorrhage (IVH) [6,7]. In contrast, vaginal delivery may offer advantages for intestinal colonization through early exposure to a favorable microbiome, thereby potentially providing protection against necrotizing enterocolitis (NEC) [8,9,10,11].

From a maternal perspective, cesarean delivery at very low gestational ages carries specific risks, including increased blood loss, postoperative infections, and a higher likelihood of placental complications or uterine rupture in subsequent pregnancies [12]. These risks must be carefully weighed against potential neonatal benefits. Accordingly, the current German S3 guideline recommends individualized decision making on delivery mode between 23 + 0 and 24 + 6 weeks [13]. In the absence of randomized controlled trials, current practice is largely based on retrospective data and clinical experience [14].

Secondary factors such as preterm premature rupture of membranes (PPROMs) and abnormal vaginal microbiota remain underexplored in this context. The PPROM is associated with infection, shortened gestation, and neonatal complications, including sepsis and NEC [15,16]. Likewise, reduced Lactobacillus dominance with increased Gardnerella, Ureaplasma, or Prevotella colonization has been linked to preterm birth, intrauterine infection, and neonatal sepsis [17,18]. A recent meta-analysis reported neonatal survival rates of about 39% following expectant management of PPROM at 14 + 0 to 25 + 6 weeks, compared to no survival after immediate delivery. The maternal risk, however, was considerable, with a maternal sepsis rate of approximately 4% and a chorioamnionitis rate of up to 30% [19]. These findings indicate that both PPROM and an altered vaginal microbiota can substantially influence neonatal outcomes and should, therefore, be taken into account when analyzing the mode of delivery.

To date, systematic analyses of these interactions, especially those below 26 weeks of gestation, are lacking. The aim of this study was therefore to evaluate neonatal outcomes in relation to delivery mode among singleton infants born between 22 + 0 and 25 + 6 weeks. Particular focus was placed on IVH, NEC, APGAR scores, and arterial cord pH, while considering prenatal risk factors such as PPROM and abnormal vaginal microbiota.

## 2. Materials and Methods

This retrospective cohort study included 92 live-born singleton infants delivered between 22 + 0 and 25 + 6 weeks of gestation between 2010 and 2021 at two university-based perinatal centers in Germany. It should be taken into account that the study covers a period of eleven years, during which neonatal treatment protocols may have changed and potentially influenced the results. Infants were eligible if delivery mode (vaginal or cesarean) and complete clinical datasets were available. Exclusion criteria were multiple gestations, intrauterine fetal deaths, and incomplete documentation.

Data was extracted from electronic medical records and standardized obstetric and neonatal documentation systems. Maternal and obstetric variables included gestational age at birth, birth weight, mode of delivery, and antenatal corticosteroid administration. Intraventricular hemorrhage (IVH) was assessed by cranial ultrasound and classified according to Papile et al. [20]. For analysis, all grades (I–IV) were summarized as “IVH any grade.” Necrotizing enterocolitis (NEC) was diagnosed according to the criteria of the German NEO-KISS registry [21]. This definition required typical clinical and radiological findings or, in the case of surgical intervention, characteristic histopathological changes. Cases corresponding only to Bell stage I were not included. Likewise, neonatal infection was defined according to the German NEO-KISS registry, requiring according to the German NEO-KISS registry, requiring with compatible clinical signs or severe and typical clinical signs in the case of culture-negative infection.

Microbiological findings from vaginal swabs (e.g., *Gardnerella*, *Enterococci*, *E. coli*) and the presence of PPROM were also recorded.

To reduce potential confounding, 1:1 propensity score matching was performed using gestational age at birth, birth weight, sex, and antenatal corticosteroid administration as matching variables. This resulted in two groups of 46 comparable cases each (vaginal vs. cesarean delivery).

Statistical analysis was conducted using Chi-square or Fisher’s exact test for categorical variables and the Mann–Whitney U test for non-normally distributed continuous variables. A *p*-value < 0.05 was considered statistically significant. For the multivariable logistic regression analysis, the outcome was defined as the occurrence of intraventricular hemorrhage (IVH, any grade) according to Papile.

## 3. Results

The median maternal age was 30 years (range 15–41) in the vaginal delivery group and 31 years (range 15–41) in the cesarean section group (*p* = 0.409). The proportion of primiparous women was identical in both groups (15.2%). Among multiparous women, a history of preterm birth was present in 84.8% of the vaginal group and 76.0% of the cesarean group (*p* = 0.500). Preterm premature rupture of membranes (PPROM) occurred in 17.4% and 21.7% of cases, respectively (*p* = 0.396). Pathological vaginal colonization was more frequent in the vaginal delivery group (76.0% vs. 52.2%; *p* = 0.021) (Table 1).

Maternal and neonatal baseline characteristics are summarized in (Table 2). The median gestational age and birth weight did not differ significantly between vaginal and cesarean deliveries. In contrast, the administration of antenatal corticosteroids (ANCS) showed a significant difference between groups (*p* = 0.021), with complete two-dose courses more common in the cesarean section group (69.6%) compared with the vaginal delivery group (39.2%). The distribution of neonatal sex was comparable between groups (*p* = 0.405).

**Figure 1:**
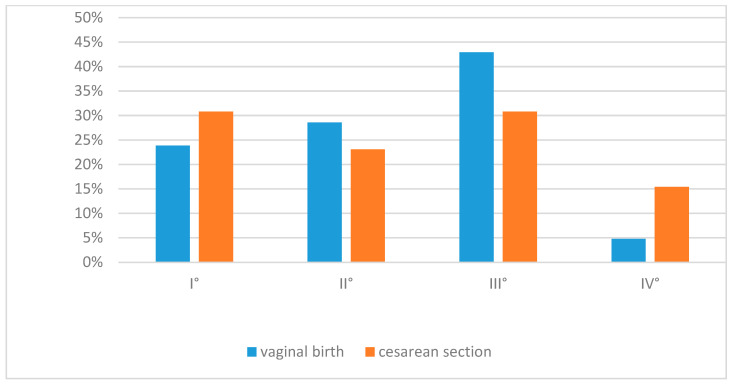
Distribution of intraventricular hemorrhage (IVH) grades (Papile I–IV) by mode of delivery. Abbreviations: IVH = intraventricular hemorrhage.

The distribution of intraventricular hemorrhage (IVH) grades according to Papile I–IV is illustrated in (Figure 1). Although IVH occurred more frequently after vaginal delivery (47.8% vs. 30.4%), this difference did not reach statistical significance (*p* = 0.080).

The APGAR scores did not differ significantly between groups. At 1 min, the median was 3 (range 0–8) after vaginal delivery and 3.5 (range 1–8) after cesarean section (*p* = 0.286). At 5 min, both groups had a median of 6 (*p* = 0.264), and at 10 min the scores were 7.5 and 7, respectively (*p* = 0.281). Arterial umbilical cord pH was comparable (median 7.35 vs. 7.32; *p* = 0.229). Survival until NICU discharge was 84.8% in the vaginal group and 93.5% in the cesarean group (*p* = 0.140). The incidence of NEC was similar (6.5% vs. 8.7%; *p* = 0.513). In contrast, IVH occurred more frequently after vaginal delivery (47.8% vs. 30.4%), although this difference did not reach statistical significance (*p* = 0.080) (Table 3).

In the multivariable logistic regression analysis, male sex was the only significant predictor of intraventricular hemorrhage (IVH, any grade) (B = –1.318; *p* = 0.041).

PPROM showed a statistical trend toward increased risk (*p* = 0.079) but did not reach significance. None of the other variables—including mode of delivery, APGAR scores, arterial cord pH, gestational age, birth weight, pathological vaginal colonization, or incomplete ANCS administration were associated with adverse outcome. The main findings of the regression model are summarized in (Table 4).

Vaginal delivery was associated with more frequent pathological vaginal colonization, while complete antenatal corticosteroid administration was significantly more common in the cesarean section group. Survival until NICU discharge and the incidence of IVH and NEC did not differ significantly between groups (Table 5).

The post hoc power analysis demonstrated low power values for all investigated endpoints (survival 26.6%, IVH 40%, NEC 5.9%), indicating that moderate but clinically relevant differences between the groups cannot be reliably detected with the present sample size (Table 6).

Pathological vaginal colonization was more frequently detected in the vaginal delivery group (76.0% vs. 52.2%). A highly significant correlation was observed between vaginal colonization and neonatal infection (Spearman’s rho = –0.344, *p* = 0.001). In contrast, no association with survival until discharge was found (Spearman’s rho = 0.013, *p* = 0.902). Regarding NEC, no significant association was detected, although a trend was noted (Spearman’s rho = –0.213, *p* = 0.052) (Table 7).

The results showed no significant differences in gestational age, birth weight, or survival until discharge between vaginal and cesarean delivery. Complete antenatal corticosteroid administration was significantly more frequent in the cesarean group (*p* = 0.021), representing a relevant confounder. IVH occurred more often after vaginal delivery (47.8% vs. 30.4%), although the difference did not reach statistical significance (*p* = 0.080). The incidence of NEC was comparable between groups, and no significant differences were observed for APGAR scores or arterial cord pH. In the multivariable regression analysis, male sex was the only factor significantly associated with adverse neonatal outcome (*p* = 0.041), whereas PPROM showed no significant association.

## 4. Discussion

The results presented in Table 5 highlight the importance of obstetric decision making in the context of extreme prematurity. In this analysis, the mode of delivery (vaginal vs. cesarean section) did not significantly affect survival until discharge from the neonatal intensive care unit, with high survival rates in both groups. This finding is consistent with current evidence suggesting that survival in extremely preterm infants depends primarily on factors such as birth weight, prenatal care, and the structural quality of neonatal intensive care [22,23].

Despite comparable survival rates, differences in neonatal morbidity were observed. There was a non-significant trend toward higher IVH rates after vaginal delivery, while NEC occurred slightly less frequently; however, neither difference reached statistical significance [9,22].

Our supplementary analysis indicated that pathological vaginal colonization was more common in the vaginal delivery group. This colonization was significantly associated with neonatal infection but showed no relation to survival until discharge. For NEC, only a non-significant trend was observed. Notably, the NEC rate was not increased in the vaginal delivery group despite the higher prevalence of pathogenic colonization. This may partly reflect differences in early microbial colonization after vaginal birth; however, our data do not provide direct evidence for such an association. The interplay between mode of delivery, vaginal microbiome, and neonatal complications, therefore, warrants further investigation in future studies (Table 7).

Conversely, mechanical stress and hemodynamic fluctuations during vaginal delivery may contribute to IVH. Some studies suggest that cesarean section may partially protect the immature brain [9,22], although the evidence remains heterogeneous. Lee et al. found no significant association between delivery mode and IVH after adjusting for gestational age and birth weight, underscoring the importance of individual risk factors and institutional standards [24]. Regional variation and differences in neonatal care infrastructure further influence outcomes in this population [25].

In comparison with previous studies that reported partly conflicting results regarding the impact of mode of delivery, our analysis adds to the existing evidence by providing a balanced perspective focusing on short-term morbidity patterns and by highlighting the role of prenatal risk factors such as PPROM and vaginal microbiota.

A key finding of our study is the imbalance in antenatal corticosteroid (ANCS) administration. In the cesarean group, 69.6% of women received two complete doses compared to 39.2% in the vaginal group. This reflects a significant confounder, as planned cesarean deliveries often allow sufficient time for full ANCS administration, whereas imminent vaginal births frequently preclude the recommended 24 h interval. The protective role of ANCS, particularly against IVH and pulmonary morbidity, is well established and may partly explain the observed group differences [26].

Several limitations must be acknowledged. The relatively small sample size of 92 singleton births between 22 + 0 and 25 + 6 weeks limits statistical power, especially for rare outcomes such as NEC and severe IVH.

One clear limitation of the present study is the small sample size. Although we combined data from two study sites, this remains a retrospective investigation. To ensure comparability, a 1:1 matching of vaginal deliveries (n = 46) and cesarean sections (n = 46) was performed. This may have led to insufficient representation of relevant differences, particularly with regard to survival, the occurrence of intestinal complications (NEC), or intraventricular hemorrhage (IVH). Table 6 presents the results of a post hoc power analysis (α = 0.05), which demonstrates a considerable risk of type II error (power for survival: 26.6%, IVH: 40%, NEC: 5.9%). This underscores the need for future studies with substantially larger sample sizes in order to reliably assess the potential protective effect of vaginal delivery with respect to intestinal complications.

Differences between primary and secondary cesarean sections were not analyzed, and the study focused only on short-term neonatal outcomes up to NICU discharge. Data on long-term neurodevelopmental outcomes were not available.

However, consideration of long-term developmental outcomes would be crucial to comprehensively assess the true impact of the mode of delivery on the life prospects of extremely preterm infants.

Moreover, the retrospective design precludes causal inference.

## 5. Conclusions

In summary, extremely preterm infants born between 22 + 0 and 25 + 6 weeks represent a highly vulnerable population. While mode of delivery did not significantly influence survival, morbidity patterns differed, with a possible lower risk of NEC after vaginal delivery and a potential protective effect of cesarean section against IVH. Other factors, including ANCS administration, vaginal microbiota, and PPROM, appear to exert a greater influence on neonatal outcome. These findings underscore the need for individualized decision making that integrates both obstetric and neonatal perspectives. To establish evidence-based recommendations, large multicenter prospective studies are required.

Future prospective multicenter studies should include stratification according to complete versus incomplete corticosteroid exposure in particular and also take long-term neurocognitive developmental outcomes into account in order to comprehensively assess the clinical relevance of different modes of delivery.

## Figures and Tables

**Table 1:** Comparison of maternal baseline characteristics by mode of delivery.

Variable	Vaginal Birth n = 46	Cesarean Section n = 46	*p*-Value
Maternal age (years)	30 (15–41) P25–P75: 25–33	31 (15–41) P25–P75: 26–35	0.409
Primiparae	7 (15.2%)	7 (15.2%)	-
Multiparae	39 (84.8%)	39 (84.8%)	0.500
Multiparae: previous birth Term birth	7 (15.2%)	12 (24.0%)	0.396
Multiparae: previous birth Preterm birth	32 (84.8%)	34 (76.0%)	0.021
PPROM	17.4% (8)	21.7% (10)	0.396
No PPROM	82.6% (38)	78.3% (36)	
Vaginal microbial colonization	35 (76.0%)	24 (52.2%)	0.021
No Vaginal microbial colonization	11 (24.0%)	22 (47.8)	

PPROM = preterm premature rupture of membranes; P25–P75 = interquartile range. Data are presented as n (%) or median (range, interquartile range).

**Table 2:** Neonatal parameters in comparison between vaginal delivery and cesarean section in preterm infants.

Parameter	Vaginal Delivery (n = 46)	Cesarean Section (n = 46)	*p*-Value
Gestational age at birth (days)	165 (154–182; 161–165–171)	166 (150–170; 164–166–168)	0.971
Birth weight (g)	595 (415–830; 555–595–663.5)	620 (335–1001; 588.8–620–706.3)	0.622
Antenatal corticosteroids (ANCS)			0.021
None	9 (19.5%)	4 (8.7%)	
One dose	19 (41.3%)	10 (21.7%)	
Two doses	18 (39.2%)	32 (69.6%)	
Sex (male/female)	26 (56.5%)/20 (43.5%)	25 (54.3%)/21 (45.7%)	0.405

ANCS = antenatal corticosteroids.

**Table 3:** Neonatal Outcomes by Mode of Delivery (Vaginal/Cesarean Section).

Variable	Vaginal Birth (n = 46)	Cesarean Section (n = 46)	*p*-Value
APGAR 1 min Median (Range) P25–P50–P75	3 (0–8) 2–3–5	3.5 (1–8) 2–3.5–5	0.286
APGAR 5 min Median (Range) P25–P50–P75	6 (0–9) 4–6–7	6 (2–9) 5–6–8	0.264
APGAR 10 min Median (Range) P25–P50–P75	7.5 (0–10) 6–7.5–8	7(4–10) 7–7–9	0.281
Umbilical arterial cord pH	7.35 (6.95–7.50) 7.285–7.30–7.39	7.32 (7.12–7.44) 7.283–7.32–7.38	0.229
Survival until NICU Discharge	Yes 39 (84.8%) No 7 (15.2%)	Yes 43 (93.5%) No 3 (6.5%)	0.140
NEC	Yes 3 (6.5%) No 43 (93.5%)	Yes 4 (8.7%) No 42 (91.3%)	0.513
IVH any	Yes 22 (47.8%) No 24 (52.2%)	Yes 14 (30.4%) No 32 (69.6%)	0.080

NICU = neonatal intensive care unit; NEC = necrotizing enterocolitis; IVH = intraventricular hemorrhage.

**Table 4:** Multivariate Logistic Regression Analysis of Perinatal and Neonatal Risk Factors for Intraventricular Hemorrhage (IVH, any grade).

Variable	Regression Coefficient (B)	Standard Error	*p* = Value
Mode of birth	–0.657	0.558	*p* = 0.239
Arterial pH	–0.236	0.275	*p* = 0.391
APGAR 1 min	–0.255	0.192	*p* = 0.185
APGAR 5 min	–0.100	0.256	*p* = 0.697
APGAR 10 min	0.047	0.252	*p* = 0.854
Pathological vaginal colonization	0.135	0.605	*p* = 0.824
PPROM	1.648	0.938	*p* = 0.079
Birthweight	–0.002	0.002	*p* = 0.486
Gestational age	0.021	0.057	*p* = 0.708
Male sex	–1.318	0.643	*p* = 0.041
ANCS (incomplete)	–0.440	0.431	*p* = 0.308
Constant term	0.062	9.030	*p* = 0.995

Outcome variable: In the analysis, the occurrence of intraventricular hemorrhage (IVH), regardless of severity, classified according to the Papile classification, served as the dependent variable.

**Table 5:** Comparison of Perinatal and Neonatal Outcomes between Vaginal Delivery and Cesarean Section.

Parameter	Vaginal Delivery (n = 46)	Cesarean Section (n = 46)	*p*-Value	Interpretation
Maternal age (years)	30 (15–41)	31 (15–41)	0.409	Comparable maternal age
Multiparity with prior preterm birth, n (%)	32 (84.8)	34 (76.0)	0.021	Higher rate after vaginal delivery
Vaginal microbial colonization, n (%)	35 (76.1)	24 (52.2)	0.021	Significantly more frequent in vaginal group
Gestational age at birth (days)	165 (154–182)	166 (150–170)	0.971	No difference
Birth weight (g)	595 (415–830)	620 (335–1001)	0.622	No difference
Antenatal corticosteroids (two doses), n (%)	18 (39.2)	32 (69.6)	0.021	More frequent in the cesarean group
Survival to NICU discharge, n (%)	39 (84.8)	43 (93.5)	0.140	Higher survival trend in the cesarean group, not significant
NEC, n (%)	3 (6.5)	4 (8.7)	0.513	Comparable incidence
IVH, n (%)	22 (47.8)	14 (30.4)	0.080	Higher after vaginal delivery, not significant

NICU = neonatal intensive care unit; NEC = necrotizing enterocolitis; IVH = intraventricular hemorrhage.

**Table 6:** Post hoc power analysis for survival until discharge, intraventricular hemorrhage (IVH), and necrotizing enterocolitis (NEC) according to mode of delivery (vaginal vs. cesarean section).

Parameter	Vaginal Birth	Cesarean Section	Power
Survival until discharge	84.8%	93.5%	26.6%
IVH	47.8%	30.4%	40.0%
NEC	6.5%	8.7%	5.9%

IVH = intraventricular hemorrhage; NEC = necrotizing enterocolitis. Power analysis was performed post hoc with α = 0.05.

**Table 7:** Association between vaginal colonization and neonatal outcomes.

Parameter	Spearman’s Rho	*p*-Wert	Interpretation
Neonatal infection	−0.344	0.001	highly significant correlation
Survival until discharge	0.013	0.902	no association
NEC	−0.213	0.052	non-significant trend/trend without statistical significance on-significant trend/trend without statistical significance

NEC = necrotizing enterocolitis. Correlations were calculated using Spearman’s rank correlation coefficient (two-tailed test).

## Data Availability

The data used and analyzed in this study are stored in the clinical database in anonymized form and cannot be made publicly available due to privacy and ethical restrictions. Upon reasonable request, the data can be provided in anonymized form by the corresponding author.
